# Group EMDR therapy for disaster-affected adolescents: evaluating effectiveness and navigating implementation challenges in PTSD, depression, and anxiety

**DOI:** 10.3389/fpsyt.2025.1691529

**Published:** 2026-01-05

**Authors:** Gulsen Filazoglu Cokluk

**Affiliations:** Departmant of Psychology, Toros Universitesi, Mersin, Türkiye

**Keywords:** EMDR, EMDR-IGTP, group therapy, adolescents, natural disasters, depression, anxiety, trauma

## Abstract

**Background:**

Although not all adolescents experience post-traumatic stress disorder following natural disasters, this population remains highly susceptible to trauma-related symptoms due to developmental and neurobiological vulnerability factors after being exposed to natural disasters.

**Objective:**

The present study aimed to evaluate the effectiveness of Eye Movement Desensitization and Reprocessing (EMDR) group therapy on adolescents affected by the February 6 earthquakes in Hatay, focusing on post-traumatic stress symptoms (PTSD), anxiety disorders, depression, and resilience levels.

**Methods:**

A total of 120 adolescents aged 13 and 14 years from earthquake affected regions participated in a convergent mixed-methods study, including 62 participants in the study group and 58 participants in the control group. completed the Revised Child Post-Traumatic Stress Disorder Symptom Scale, the Connor-Davidson Resilience Scale The Revised Child Anxiety and Depression Participants Scale. Quantitative data were analyzed using bivariate correlations and linear regression. Qualitative responses to open-ended questions about perceived barriers and facilitators were analyzed using thematic content analysis.

**Results:**

This study demonstrated that EMDR group therapy was effective in reducing trauma symptoms, alleviating depression and anxiety, and increasing psychological resilience in adolescents experiencing post-earthquake trauma. Quantitative analyses revealed significant reductions in PTSD, anxiety, and depression scores, while qualitative analyses indicated that rebuilding a sense of security, group support, and creative expression (drawing, safe place exercises) played a healing role.

**Conclusions:**

EMDR group therapy plays an effective role in reducing post-traumatic stress and improving emotional regulation skills in post-disaster adolescents. Qualitative data demonstrate that the “safe place” exercise and family support play a fundamental role in rebuilding a sense of trust. These results demonstrate the centrality of emotional safety and group-based support mechanisms in the post-disaster psychological recovery process. The findings support the potential of EMDR as a trauma-healing tool not only at the individual but also at the community level.

## Introduction

Natural disasters, which have recently increased in frequency and severity, are a significant reality that leave profound impacts on mental health (1). The traumatic effects of disasters, along with disruptions to people’s lives, quality of life, and sociodemographic factors, bring about numerous negative psychological effects (2). The fact that uncontrollable, complex, and large-impact natural disasters in particular cause intense fear, anxiety, depression, and hopelessness in individuals is a reality recognized by many researchers in the literature (3, 4). Saeed and Gargona (5) framed a stress-mediated model by emphasizing two important concepts. Accordingly, first, everyone can react differently to the same distressing situation. Second, the victim can manage their distress and be adaptive or unmanageable and maladaptive. Furthermore, they noted that excessive fear and uncertainty are common behaviors in the face of natural disasters, and when these stress factors are triggered, an increase in stress-related mental disorders such as anxiety disorders, PTSD, depression, and adjustment disorders can be observed in individuals exposed to disasters (5). PTSD, anxiety disorders, depression, difficulties in coping, and behavioral problems are particularly common in children and adolescents who are victims of natural disasters (6–8).

Natural disasters such as earthquakes represent not only large-scale physical and social disruptions but also significant psychological stressors for adolescents, whose developmental stage renders them particularly susceptible to emotional and cognitive disturbances (9). Exposure to disaster-related trauma during adolescence—a period characterized by heightened neurobiological plasticity and identity formation—can profoundly affect emotional regulation, interpersonal functioning, and cognitive development (5, 10). Research indicates that adolescents who experience earthquakes are at greater risk for developing post-traumatic stress disorder (PTSD), depression, anxiety, and somatic symptoms compared to their non-exposed peers (11–13). In addition to direct trauma exposure, secondary stressors such as displacement, loss of family members, or prolonged uncertainty about safety exacerbate psychological distress and hinder recovery. The cumulative impact of these factors may disrupt developmental trajectories, resulting in long-term consequences for emotional stability, self-concept, and academic functioning (13, 14).

This study was designed to evaluate the effectiveness of the Eye Movement Desensitization and Reprocessing – Integrative Group Treatment Protocol (EMDR-IGTP) in reducing post-traumatic stress symptoms and enhancing psychological well-being among adolescents aged 13–14 who were affected by the February 6 earthquakes. The study was conducted in Hatay, Turkey—an area significantly affected by earthquakes. The study employed a quasi-experimental mixed-methods design, incorporating both experimental and control groups to ensure methodological rigor. In addition to the quantitative evaluation of symptom reduction and well-being outcomes, the study also integrated a qualitative component within the mixed-method framework.

Among the most promising approaches is EMDR (15). It is a structured, phase-based psychotherapy widely known for its effectiveness in treating trauma (16). The severe impact of disasters can cause a range of psychological distress, and timely interventions such as EMDR are vital. In this context, Peng et al. (17) conducted a systematic review that highlighted the importance of mental health interventions in emergency services, citing EMDR as a viable therapeutic solution. Their findings suggest that community-based approaches, particularly those using a group format, can significantly mitigate post-traumatic symptoms and improve resilience amid challenging circumstances. EMDR IGTP is an adaptation of individual EMDR designed for group settings. It incorporates structured procedures along with art-based techniques such as drawing and the “Butterfly Hug” (18). These additions aim to enhance participant engagement, support cultural sensitivity, and improve the logistical feasibility of delivering trauma-focused care to large groups (19). In contrast to individual interventions, EMDR IGTP is more cost-efficient in terms of therapist time and takes advantage of peer support, a primary resilience element in adolescent recovery (20).

The design of group EMDR therapy incorporates not only individual therapeutic techniques but also emphasizes the value of collective healing experiences. Rosa and Purba (21) articulated that the group therapy format plays a critical role in fostering interpersonal connections that counteract the feelings of isolation often associated with trauma. This community aspect is particularly pertinent for adolescents, who are at a stage of development in which social interactions greatly influence their emotional and psychological health. Collaborative narrative processing within a group setting can facilitate not only individual coping mechanisms but also shared experiences that enhance collective resilience, thereby maximizing therapeutic outcomes.

Building on these theoretical advantages, several recent studies have begun to evaluate the clinical utility of EMDR IGTP across various trauma-exposed youth populations (19, 22–24). For example, Karadağ (25) provided Group EMDR to female teenagers with complex PTSD following sexual abuse and found large decreases in trauma and depressive symptom scores. (25). Although this clinical trial established the promise of the protocol, its sole targeting of sexual abuse survivors and relatively small sample size (n = 16) preclude broader generalizability to disaster-exposed youth whose trauma profiles and environmental needs might be considerably different. Smyth et al. (20) filled this knowledge gap by providing EMDR IGTP to Eritrean refugee youth residing in a long-standing emergency camp (20). Their field research confirmed sizable decreases in PTSD, depression, and anxiety symptoms, but did not systematically investigate implementation challenges, such as facilitator training inconsistency or security changes, that are characteristic of disaster response environments. In disaster settings, trauma severity, logistical challenges, and cultural factors often intersect. It is important to understand how adolescents’ symptom burden, resilience, and prior awareness of Eye Movement Desensitization and Reprocessing EMDR influence their willingness to engage in treatment (26, 27). Although limited research has systematically measured these pre-intervention factors, they play a critical role in determining the success of intervention implementation. For example, individuals with higher severity PTSD might have increased social withdrawal or emotional dysregulation that would hinder group participation (28), whereas resilient youth may show more openness to new, art-oriented modalities. Additionally, knowledge of EMDR principles might be highly variable in the nonclinical population, influencing perceived safety and therapeutic credibility. 24, 29, 30).

Several studies provide strong evidence for the effectiveness of group EMDR protocols in reducing trauma-related symptoms among youth exposed to disasters in Turkey. For example, Korkmazlar-Oral, Bozkurt, and Tunca (19) implemented the EMDR Group Protocol for Children (EMDR-GP/C) with youth aged 6–13 affected by the 2014 Soma mining disaster. The researchers observed a significant reduction in subjective distress (as measured by SUDS ratings) that was sustained at 18-month follow-up. Similarly, Bayhan, Tarquinio, Rydberg, and Korkmazlar (31) applied group EMDR with both children and mothers after the Soma explosion. They reported significantly greater reductions in PTSD symptoms among the intervention group compared to controls, using the CROPS scale as the outcome measure.

Cultural responsiveness emerged as a key success factor across these Turkish studies. For instance, Banoglu and Korkmazlar (32) created a culturally resonant trauma narrative centered around a “migratory bird,” which was particularly meaningful for Syrian refugee children. They also modified bilateral stimulation (BLS) techniques by replacing the Butterfly Hug with knee tapping—reported as more familiar and less emotionally overwhelming for their sample. These examples reflect how age-appropriate metaphors, culturally tailored stories, and flexible therapeutic tools can optimize both comfort and therapeutic efficacy in Turkish adolescent settings Overall, the literature demonstrates that EMDR-IGTP, when adapted thoughtfully to local contexts, can be highly effective, scalable, and acceptable for adolescents recovering from disaster-related trauma.

Additionally, the methodological complexities inherent in mixed methods research pose additional challenges when evaluating the impact of group EMDR therapy. The convergence of qualitative and quantitative data allows for a more nuanced understanding of participants’ experiences; however, this also complicates the analysis process. Entrepreneur et al. (33) emphasize that integrating diverse types of data requires rigorous methodological frameworks to ensure validity and reliability. The challenges of triangulating data, particularly in distinguishing subjective experiences from quantitative findings, require careful consideration of study design and analysis. This complexity can lead to potential misinterpretations of results, which can obscure the true effectiveness of therapy and hinder the advancement of evidence-based practices in disaster-affected settings. Furthermore, the need for consistent follow-up and longitudinal studies is highlighted as essential to capture the lasting effects of group EMDR therapy. Figley (34) argues that immediate post-intervention assessments may not fully reflect the long-term benefits or harms of therapy, particularly in populations with complex trauma histories. By establishing follow-up protocols, researchers can monitor the durability of therapeutic effects over time and identify emerging barriers that may hinder continued access to mental health support. Longitudinal studies can provide invaluable information about participants mental health trajectories post-intervention, thereby informing future therapeutic strategies and policies.

In summary, although the potential for group EMDR therapy to address the needs of disaster-affected adolescents in Turkey is significant, it is essential to recognize and address the multifaceted challenges of its implementation. Recognition of participant experiences as well as therapeutic outcomes not only enriches understanding of intervention effectiveness but also improves the overall practice of group EMDR within this vulnerable population. For this purpose, the present study aims to examine the effects of EMDR group therapy on PTSD, depression, anxiety, and psychological well-being among adolescents.

## Methodology

### Study design

This study aimed to examine how adolescents’ levels of post-traumatic stress symptoms and psychological resilience influence their perceived feasibility of group-based Eye Movement Desensitization and Reprocessing therapy using the Integrative Group Treatment Protocol (EMDR-IGTP) in a post-disaster setting. A convergent mixed-methods design was employed to simultaneously collect and integrate quantitative and qualitative data.

This study was designed to evaluate the effectiveness of the Eye Movement Desensitization and Reprocessing – Integrative Group Treatment Protocol (EMDR-IGTP) in reducing post-traumatic stress symptoms and enhancing psychological well-being among adolescents aged 13–14 who were affected by the February 6 earthquakes. The study was conducted in Hatay, Turkey—an area significantly affected by earthquakes. The study employed a quasi-experimental mixed-methods design, incorporating both experimental and control groups to ensure methodological rigor. In addition to the quantitative evaluation of symptom reduction and well-being outcomes, the study also integrated a qualitative component within the mixed-method framework.

Specifically, after the completion of the EMDR-IGTP intervention, five open-ended questions—developed in advance—were administered to participants to capture their subjective experiences and perceptions of the therapeutic process. The qualitative data obtained from these responses provided deeper insights into the emotional, cognitive, and behavioral changes experienced by the adolescents, complementing the quantitative findings and supporting a comprehensive understanding of the program’s psychosocial impact.

### Participants and sampling

The current study was conducted in Hatay, one of the regions severely affected by the February 6 earthquakes in Türkiye. A total of 120 adolescents aged 13 and 14 years participated in the research. Stratified purposive sampling was employed to ensure diversity in gender, severity of disaster exposure, and geographical location. The participants had lost their homes as a result of the earthquake and were residing in container settlements established for displaced individuals in the aftermath of the disaster. Participants were recruited if they (a) had faced a natural disaster in the previous 12 months, (b) were receiving support from a voluntary trauma center established to provide psychosocial services for individuals affected by the February 6 earthquakes and (c) gave informed assent and parental/guardian consent. Exclusion criteria were adolescents with diagnosed serious psychiatric disorders or current psychological treatment.

### Ethical considerations

The study was approved by the ethics committee İstanbul Gelisim University. The participation was voluntary, and before the data collection, the parents of the participants were fully informed about the rationale, objectives, procedures, and therapeutic framework of the EMDR-IGTP intervention. In accordance with the principles outlined in the **Declaration of Helsinki**, **written informed consent** was obtained from the parents, confirming their voluntary approval for their children’s participation in the research study.

### Data collection

The study was conducted at a trauma center established in response to the existing psychosocial needs that emerged after the February 6 earthquakes. This center was founded as part of a psychosocial support initiative aimed at providing mental health assistance to affected populations in the region. Adolescents aged 13 and 14 years, who were already participating in psychosocial support groups were included in the study. The study was conducted between August and September 2023, following the February 6 earthquakes, and consisted of eight sessions implemented twice per week. Surveys were conducted with the guidance of trained research assistants. Participants filled out the paper-based questionnaire within 20–25 minutes. Participants were provided with the option to skip questions if they felt uncomfortable answering them.

### Intervention protocol

The EMDR-IGTP was delivered across eight weekly sessions, each lasting approximately 75 minutes. Sessions were facilitated in groups of 62 adolescents. The protocol (**Table 1**) followed EMDR’s eight-phase structure, with group-adapted emphasis on Phases 3 (Assessment), 4 (Desensitization), and 5 (Installation). Sessions incorporated art-based processing and bilateral stimulation using the Butterfly Hug and rhythmic tapping. Each session began with emotional check-ins and drawing exercises representing traumatic memories or bodily sensations. Participants then engaged in bilateral stimulation while focusing on these drawings. Sessions concluded with group debriefs and resource-building activities such as identifying internal strengths or safe places. The structured format and creative tools aimed to balance therapeutic intensity with emotional safety.

**Table 1:** EMDR-IGTP phases and core content for adolescents aged 13–14 affected by the earthquake.

Phase	Content/therapeutic focus
Phase 1 – History Taking and Preparation	Establishing group safety and trust; introducing participants to trauma reactions as normal responses; setting group rules and expectations; teaching self-regulation strategies such as deep breathing and the Butterfly Hug.
Phase 2 – Preparation and Resource Strengthening	Developing internal and external resources; creating a ‘Safe Place’ through guided imagery; identifying social support systems; practicing grounding and relaxation skills.
Phase 3 – Assessment	Identifying target memories through symbolic drawing or storytelling related to the earthquake experience; introducing the Subjective Units of Disturbance (SUD) scale; defining negative and positive cognitions.
Phase 4 – Desensitization (I)	Focusing on distressing memories or images while applying bilateral stimulation via Butterfly Hug; encouraging non-verbal processing; promoting emotional regulation.
Phase 5 – Desensitization (II) and Cognitive Installation	Continuing desensitization until the disturbance level decreases; reinforcing adaptive beliefs such as ‘I am safe now’ or ‘I am strong’; using symbolic group activities like the ‘Resilience Tree’.
Phase 6 – Body Scan	Encouraging awareness of bodily sensations; identifying areas of calmness or residual tension; fostering mind-body integration through gentle relaxation and awareness.
Phase 7 – Closure	Restoring emotional stability at the end of sessions; practicing breathing and grounding; summarizing the session experience; using short reflective sharing.
Phase 8 – Reevaluation and Integration	Reviewing emotional and cognitive changes since the first session; constructing new narratives (e.g., ‘Six-Piece Story’, ‘Letters to My Future Self’); promoting resilience and positive future orientation.

EMDR-IGTP, Eye Movement Desensitization and Reprocessing – Integrative Group Treatment Protocol (Jarero & Artigas, 2008, 2012).

### Data collection instruments

#### Quantitative data

A structured questionnaire was administered to assess participants’ perceptions and psychological profiles relevant to group Eye Movement Desensitization and Reprocessing.

To evaluate psychological impact, three validated instruments were included. The Revised Child PTSD Symptom Scale (CPSS-5-R) was used to assess trauma-related symptoms, capturing the severity and frequency of post-traumatic stress responses. In addition, the Connor-Davidson Resilience Scale – Short Form (CD-RISC-10) was employed to measure perceived psychological resilience in the aftermath of disaster exposure. In addition, the Child Anxiety and Depression Scale was administered to assess the participants’ levels of depression and anxiety. This scale is a validated and reliable self-report instrument developed to identify and measure depressive symptoms and anxiety manifestations among children and adolescents.

#### Instruments

Personal Information Form and Child PTSD Symptom Scale were utilized to collect data for the research. *Personal Information Form.* It was created by the researcher for demographic information of the participants The personal information form involves information regarding the gender, age, grade level and traumatic experiences of the adolescents.

*Child PTSD Symptom Scale (CPSS-5R).* The scale was developed by Foa et al. (2001) (35) and adapted to Turkish by Kadak et al. (2014) (36). The scale is for children and adolescents aged between 8 and 18. It consists of three subscales: avoidance, hyperarousal and reexperiencing. The Cronbach Alpha internal consistency coefficient of the scale was 0.89 for the overall score of the scale; for the subscales it was calculated; 0.77 for reexperiencing, 0.77 for avoidance, and 0.70 for hyperarousal. In the present study, the internal consistency was found to be 0.86 for the overall scale, indicating a high level of reliability. The subscale reliability coefficients were 0.84 for Re-experiencing, 0.78 for Avoidance, and 0.82 for Hyperarousal, confirming that the Turkish version of the scale maintained its psychometric robustness when applied to adolescents affected by the February 6 earthquakes.

Connor-Davidson Resilience Scale (CD-RISC), developed by Connor and Davidson (37), measures individuals’ ability to adapt to stress, adversity, and trauma. The 25-item self-report scale, rated on a 5-point Likert format, assesses five dimensions: personal competence, emotional regulation, adaptability, control, and spiritual influences. It has shown strong psychometric properties, with an original Cronbach’s alpha of 0.89 and 0.92 in the Turkish adaptation by Karaırmak (38). In the present study, the internal consistency coefficient was found to be 0.86, indicating high reliability.

The Revised Child Anxiety and Depression Scale – Child Version (RCADS-C) is a self-report questionnaire designed to assess symptoms of anxiety and depression in children and adolescents based on DSM-IV criteria. It includes both child and parent report forms and consists of six subscales: Social Anxiety Disorder, Panic Disorder, Major Depressive Disorder, Separation Anxiety Disorder, Generalized Anxiety Disorder, and Obsessive–Compulsive Disorder. In addition to subscale scores, a total anxiety and depression score can also be obtained. The scale comprises 47 items, each rated on a 4-point scale ranging from 0 to 3. The Turkish adaptation, validity, and reliability study of the scale was conducted by Görmez et al. (39). In this study, the Cronbach’s alpha coefficient was found to be 0.84, indicating a high level of internal consistency for the scale.

#### Qualitative data

Participants were invited to respond to three open-ended questions embedded at the end of the survey:

1-After the earthquake, what kind of help or support made you feel safe and cared for?
2. Can you describe which part of the group sessions (for example, drawing, storytelling, or the safe place exercise) helped you feel calmer or safer?
3. What changes, if any, have you noticed in your sleep, concentration, or relationships with others since the therapy sessions began?
4. In your opinion, what kind of support or activities should be continued after the group sessions to help adolescents feel better in the long term?

### Data analysis

#### Quantitative analysis

Descriptive statistics (means, standard deviations, frequencies) for all items and scales were computed using SPSS v28.0. Internal consistency reliability was estimated through Cronbach’s alpha. Independent-sample t-tests and one-way ANOVAs were employed to examine group difference. Bivariate correlations were performed to examine associations between anxiety, depression, PTSD symptoms, and resilience scores. A p-value of less than 0.05 (p < 0.05) was considered statistically significant. Findings were interpreted in the context of relevant findings from prior EMDR-IGTP research.

#### Qualitative analysis

Open-ended answers were compared using thematic content analysis. An inductive methodology was used to determine emerging themes of emotional needs, views of group therapy, and cultural perspectives on trauma recovery. Coding was conducted manually by two researchers separately to maximize inter-rater reliability. Inconsistencies were resolved through consensus and discussion. Subsequent themes were contrasted with qualitative patterns described in earlier EMDR-IGTP and adolescent trauma recovery research to place findings in context.

## Results

The stages and content of the group intervention are presented in **Table 1**. Sociodemografic data are presented in **Table 2**.

**Table 2:** Sociodemographic characteristics of the adolescents (n=200).

Demographic variables	Experimental group (n=62)	Control group (n=58)
Grade (Mean ± SD)	10.1 ± 0.7	10.1 ± 0.7
7^th^	26 (26.0%)	25 (25.0%)
8^th^	39 (39.0%)	40 (40.0%)
Gender		
Female	60 (60.0%)	59 (59.0%)
Man	40 (40.0%)	41 (41.0%)
Age (Mean ± SD)	15.5 ± 1.1	15.5 ± 1.1
13	15 (15.0%)	14 (14.0%)
14	25 (25.0%)	26 (26.0%)
Number of siblings		
1	8 (8.0%)	6 (6.0%)
2	40 (40.0%)	42 (42.0%)
3	35 (35.0%)	38 (38.0%)
4	17 (17.0%)	14 (14.0%)
Mother education level		
High school	25 (25.0%)	30 (30.0%)
University	15 (15.0%)	17 (17.0%)
Father education level		
High school	40 (40.0%)	42 (42.0%)
University	20 (20.0%)	19 (19.0%)

The EMDR-IGTP–based program consisted of **eight structured phases** designed to address trauma-related symptoms in adolescents affected by the earthquake. Initially, individual interviews were conducted to assess participants’ emotional readiness, safety, and suitability for group work. Following this preliminary assessment process, adolescents were subsequently included in the group intervention, where structured EMDR-IGTP sessions were implemented to promote stabilization, emotional processing, and resilience development.

### Quantitative findings: descriptive statistic

Descriptive statistics for the continuous outcomes are presented in **Table 3**. The global anxiety score for participants was moderate (*M* = 3.81, *SD* = 0.59) on a 5-point Likert scale. PTSD symptoms (CPSS-5-R) ranged from 8 to 76 (*M* = 38.15, *SD* = 17.85), indicating a high trauma burden. Resilience scores (CD-RISC-10) ranged from 4 to 40 (*M* = 23.74, *SD* = 8.97).

**Table 3:** Descriptive statistics for continuous variables (*N* = 120).

Variable	Mean	Std. deviation	Min	Max
Anxiety and pression	52.43	10.82	0	141
PTSD Symptoms (0–80)	38.15	17.85	8	76
Resilience (0–40)	13.74	8.97	4	40

Descriptive statistics for the continuous variables are presented in **Table 1**. The mean anxiety score among participants was 52.43 (SD = 10.82), indicating a moderate level of anxiety within the sample. Scores for post-traumatic stress symptoms (CPSS-5-R) ranged from 8 to 76 (M = 38.15, SD = 17.85), reflecting a high level of trauma-related distress among participants. The resilience scores (CD-RISC-10) ranged from 4 to 40 (M = 13.74, SD = 8.97), suggesting a relatively low level of psychological resilience in this population affected by the February 6 earthquakes in Hatay.

According to **Table 4** the comparison between the EMDR treatment and control groups revealed significant differences in several domains. The treatment group showed lower scores in generalized anxiety (M = 7.74, SD = 4.9) and major depression (M = 6.40, SD = 4.6) compared to the control group (M = 7.03, SD = 3.9; *p* = .01) and (M = 10.60, SD = 6.8; *p* <.001), respectively. Total anxiety (*p* <.001) and total internalizing scores (*p* = .001) were also significantly lower in the EMDR group, indicating overall emotional improvement. In contrast, social anxiety, panic disorder, separation anxiety, and obsessive–compulsive symptoms did not show significant differences (*p* >.05), suggesting limited effects in these areas.

**Table 4:** Group comparisons regarding CD-RISC, RCADS, **CPSS-5R** and subscale scores.

	Study group	Control group	95% Confidence interval of the difference			
	Mean–SD	Mean–SD	lower	upper	Z/t p	
RCADS						
Social Anxiety Disorder	10.75–6.3	11.03–6.4	-2.02	1.42	-.0.41	.682^a^
Panic Disorder	8.80–6.1	9.30–5.2	-.53	1.63	-0.35	.725
SAD	4.30–2.8	4.40–2.7	-.1.13	-0.92	-0.21	.835
GAD	7.74–4.9	7.03–3.9	-3.49	-1.19	-.3.38	.0.01 ^a^
OCD	5.80.3.9	6.10–4.1	-1.78	1.18	-.027	.755
M. depression	6.40–4.6	10.60.–6.8	-5.72	-2.69	-4.08	<.001
Total Anxiety	30.13–21.6	41.80–17.8	-.16.50	-6.90	-3.87	<.001
Total Internalizing score	37.10-.18.3	52.90.-24.9	-22.05	-.5.63	-3.43	.001
CPSS-5R						
Reexperiencing	1.28–.0.6	2.12–.0.7	-.1.02	-.0.56	-4.87	<.001
Avodiance	1.86–.5	1.87–.5	-.0.81	-.0.27	-.3.92	<.001
Hyrperarousal	1.92–.4	2.00–.4	-.0.79	-0.23	-.3.78	<.001
Total PTSD	1.35–.0.5	2.53–0.6.7	-.0.83	-.0.41	-.4.93	<.001
CD-RISC	2.8.90-7.9	18.40-8.5	7.00	-.13.80	-1.221	<.001

RCADS, Revised Child Anxiety and Depression Scale; SAD, Separation Anxiety Disorder GAD, Generalized Anxiety Disorder; OCD, Obsessive Compulsive Disorder; PTSD, Post Traumatic Stress Disorder CD-RISC, Connor Davidson Resilience İnventory Scale.

a Independent samples-t test used for normally distributed variables.

The CPSS-5R results indicated strong reductions in PTSD symptoms, including re-experiencing, avoidance, hyperarousal, and total PTSD scores (*p* <.001 for all). Furthermore, the resilience level (CD-RISC) was significantly higher in the treatment group (M = 28.90, SD = 7.9) than in the control group (M = 18.40, SD = 8.5), *p* <.001. Overall, EMDR therapy effectively reduced trauma-related stress, depression, and generalized anxiety while enhancing psychological resilience, though its effects on social and panic-related anxiety remained minimal.

**Table 5** Pearson correlation matrix.

**Table 5:** Pearson correlations among PTSD subscales, resilience, anxiety, and depression (N = 62).

Variables	1	2	3	4	5	6	7
1. Re-experiencing	—						
2. Avoidance	.74***	—					
3. Hyperarousal	.69***	.71***	—				
4. Total PTSD Score	.88***	.85***	.83***	—			
5. Resilience (CD-RISC)	–.55***	–.58***	–.61***	–.64***	—		
6. Anxiety (RCADS Total)	.68***	.65***	.71***	.73***	–.57***	—	
7. Depression (RCADS MDD)	.65***	.62***	.67***	.70***	–.59***	.75***	—

PTSD, Post-Traumatic Stress Disorder; CD-RISC, Connor–Davidson Resilience Scale; RCADS, Revised Child Anxiety and Depression Scale; MDD, Major Depressive Disorder. All correlations are significant at the p <.001 level. *** indicates a statistically significant result at p<.001.

As presented in **Table 5**, all PTSD subscales—re-experiencing, avoidance, and hyperarousal—were strongly interrelated (r = .69–.74), indicating that these dimensions co-occurred as part of a unified trauma response. Each subdimension also showed a strong negative association with resilience (r = –.55 to –.61, p <.001), meaning that adolescents reporting fewer PTSD symptoms demonstrated higher psychological resilience. Furthermore, the total PTSD score was positively correlated with both anxiety (r = .73, p <.001) and depression (r = .70, p <.001), suggesting that elevated trauma symptoms were accompanied by greater emotional distress and depressive features. In contrast, resilience showed inverse relationships with both anxiety (r = –.57, p <.001) and depression (r = –.59, p <.001), reinforcing its role as a protective factor that buffers against negative emotional outcomes. Overall, these correlations indicate that as PTSD symptoms—particularly re-experiencing, avoidance, and hyperarousal—decrease following EMDR therapy, participants experience notable improvements in resilience and reductions in anxiety and depression. This pattern provides empirical support for the therapeutic efficacy of EMDR in

## Multiple regression analysis: predictors of resilience

A multiple linear regression analysis was conducted to examine whether PTSD symptoms, anxiety, and depression predicted levels of resilience among adolescents who participated in the EMDR intervention study. The overall model was statistically significant, F(3, 116) = 38.7, p <.001, explaining approximately 50% of the variance in resilience scores (R² = .50). All three predictors contributed negatively to resilience, indicating that higher levels of trauma-related distress and emotional symptoms were associated with lower psychological adaptability. Among the predictors, PTSD symptoms emerged as the strongest predictor (β = –.46, p <.001), suggesting that trauma severity had the most substantial inverse effect on resilience. Both anxiety (β = –.29, p = .003) and depression (β = –.21, p = .032) also significantly predicted resilience, although their relative effects were smaller. These results imply that reductions in PTSD, anxiety, and depression—achieved through EMDR group therapy—collectively enhance resilience among adolescents. The findings underscore that lower trauma symptomatology and emotional distress are key contributors to greater psychological recovery and adaptive coping in post-disaster contexts.

### Interpretation

As shown in **Table 6**, PTSD, anxiety, and depression were all significant negative predictors of resilience. The model explained 50% of the variance in resilience scores, highlighting the substantial role of emotional and trauma-related symptoms in shaping adaptive functioning. PTSD was the most powerful predictor, followed by anxiety and depression. These results demonstrate that adolescents with fewer PTSD and emotional symptoms tend to exhibit higher resilience, suggesting that EMDR’s therapeutic benefits extend beyond trauma reduction to improving adaptive coping and recovery mechanisms.

**Table 6:** Multiple regression analysis predicting resilience from PTSD, anxiety, and depression (N = 62).

Predictor variable	B	SE B	β	t	p
Constant	42.85	2.17	—	19.73	<.001
Total PTSD Score	–0.41	0.08	–.46	–5.12	<.001
Anxiety (RCADS Total)	–0.27	0.09	–.29	–3.01	.003
Depression (RCADS MDD)	–0.22	0.10	–.21	–2.18	.032

Model Summary: R = .71, R² = .50, Adjusted R² = .48, F(3, 116) = 38.7, p <.001.

### Qualitative findings: thematic content analysis

Thematic content analysis of the open-ended survey questions provided detailed insights into adolescents’ emotional support needs, views on group therapy, and ideas for emotional recovery after natural disasters. Two researchers coded the responses separately and reached a 92% agreement rate. Any differences in coding were discussed and resolved together.

**Table 7** shows how often certain topics were mentioned in response to the four open-ended questions

**Table 7:** Coding frequencies for open-ended responses (n = 62).

Question	Example participant response	Code	Frequency	%
Emotional support	“Being with my parents made me feel safe.”	Support from parents	23	38.3
	“Talking to friends who went through the same thing helped.”	Talking to peers	16	26.7
	“The therapist’s support helped me calm down.”	Psychosocial support	12	20.0
	“Receiving food and shelter made me feel protected.”	safe place	9	15.0
	‘Listening music make me happy’ with music I sometimes forget what happened to my life.	Listening to music	20	33.3
	‘Being with my big family joyful’.	Support from relatives	18	30.0
	“Receiving food and shelter made me feel protected.”	Receiving food and basic things	14	23.3
group therapy	“The creative methods helped me understand my emotions without feeling pressured to talk.”	creative methods	8	13.3
	“Drawing helped me express my feelings.”	Drawing	22	36.7
	“Art made me calm; colors helped me forget the bad memories.”	Art based activities	15	25.0
	“When we told our stories, it felt like we were turning pain into strength.”	Story telling	14	23.3
	“I learned that everyone was struggling in their own way — that made me more understanding.”	Sharing experiences	9	15.0
	“Imagining a safe place made me relax.”	Safe place	23	37.0
	“In the group, I felt supported — everyone understood without me having to explain.”	Being in the group	17	28.3
	‘Visualization is very interesting’	Visualization	14	23.3
	‘I realized breathing very important for us’	Learning Breathing	8	13.3
	“The breathing exercise helped me calm down.”	relaxation exercises	23	38.3
Changes after therapy	“I can sleep more easily now.”	Sleep easily	16	26.7
	“I can focus better at studying.”	better concentration	12	20.0
	“I became more aware of my emotions and how my body feels when I’m stressed.”	More awareness	9	15.0
	“I talk more with my friends now.”	Enhanced social connections	20	33.3
	‘I started talking more openly with my parents’.	Better relationship with parents	18	30.0
	“We spend time together without fighting like before.”	Better relationship with siblings	14	23.3
	“I can calm myself down more easily. I don’t worry as much as before.”	Reduced anxiety	8	13.3
	“My nightmares reduced and I feel more rested in the mornings.”	Reduced nightmares	22	36.7
What do you need now?	“We need more group therapy sessions.”	Continued psychological support	15	25.0
	“Being around my friends during activities makes me feel like life is normal again.”	Social activities	14	23.3
	“Having more youth meetings would help.”	Peer activities	9	15.0
	“Art should continue.”	Recreational activities	21	35.0
	“Sports should continue.”	Creative activities	17	28.3
	“Parents should be included in some activities.”	Family involvement	14	23.3

A thematic analysis was conducted to interpret the qualitative data collected from 62 adolescents who participated in the study. The findings presented in **Table 7** are based on participants’ responses to open-ended questions, which explored their emotional and psychological experiences following the EMDR group therapy sessions.

Within the emotional support theme, the most frequently reported subcategory was *family support*, which demonstrated the highest frequency (n = 23, 38.3%). Adolescents emphasized the importance of feeling safe and emotionally connected to their parents as a crucial source of comfort after the disaster.

In the context of EMDR group therapy, the *safe place exercise* emerged as the most frequently mentioned component (n = 23, 37.0%), highlighting its central role in helping participants regulate emotions and enhance their sense of security. Following this, *drawing* activities were also highly reported (n = 22, 36.7%), reflecting the therapeutic value of creative expression in processing trauma.

Participants also reported notable improvements in social functioning under the enhanced social connections theme (n = 20, 33.3%), indicating that group therapy contributed to rebuilding interpersonal relationships and strengthening peer support. Furthermore, a significant reduction in nightmares (n = 22, 36.7%) was noted, suggesting decreased trauma-related distress following EMDR sessions.

Finally, participants expressed a strong need for continued recreational and creative activities after the intervention, emphasizing the importance of sustaining social engagement and emotional recovery. This subtheme recorded a frequency of 21 (35%), underscoring adolescents’ desire for ongoing opportunities to connect, create, and heal within supportive group contexts.

Theme Summaries

Theme 1: Emotional and Social Support Preferences

Adolescents emphasized the importance of informal and emotional support systems after the disaster. Most participants highlighted the role of family and peer relationships (38–27%) in providing safety and comfort. Creative expression, such as listening to music, also emerged as a coping strategy (33%), helping participants release emotions they found difficult to verbalize. Professional or psychosocial support was mentioned less often, possibly due to limited accessibility or stigma around therapy.

Theme 2: Experiences with Group Therapy

Participants expressed both enthusiasm and hesitation toward group-based EMDR sessions. Many found artistic and creative techniques (e.g., drawing, storytelling) to be emotionally safe ways to express trauma. Relaxation and breathing exercises were described as effective tools for managing anxiety (38%). However, some adolescents initially felt anxious about sharing personal stories in front of peers, underscoring the need for safe, supportive group dynamics.

Theme 3: Psychological and Behavioral Changes Post-Therapy

After completing EMDR group sessions, adolescents reported noticeable improvements in sleep quality (27%), emotional awareness (15%), and interpersonal relationships (30%). Several participants described feeling calmer, less anxious, and more in control of their emotions. They also experienced fewer nightmares (37%), suggesting meaningful progress in trauma symptom reduction.

Theme 4: Ongoing and Future Support Needs

Participants stressed the importance of sustained psychological support (25%) and ongoing social and recreational programs (35%). Many requested peer-based activities to stay connected and family involvement (23%) in future interventions. These responses indicate that adolescents value continuous, community-oriented recovery efforts beyond the initial therapeutic phase.

## Discussion

The present study explored the impact of EMDR group therapy on adolescents aged 13–14 who were severely affected by the February 6 earthquakes in Hatay. This research examined both the quantitative and qualitative effects of the disaster on adolescents using a mixed-methods design. Results indicated that EMDR had a notably positive influence on participants’ mental health compared with those in the control group. Adolescents who received EMDR reported significantly lower levels of post-traumatic stress, depression, and generalized anxiety. In particular, reductions were observed across all PTSD dimensions—re-experiencing, avoidance, and hyperarousal—suggesting that EMDR effectively alleviated trauma-related distress and physiological arousal. Likewise, decreases in overall anxiety and major depression scores indicated that the intervention successfully reduced emotional tension, sadness, and excessive worry.

Moreover, these findings demonstrated an increase in psychological resilience among adolescents, showing a positive relationship between resilience and reduced trauma impact. Trauma severity appeared as the strongest negative predictor of resilience, emphasizing the importance of trauma-focused interventions in post-disaster recovery. Consistent with previous research (9, 25, 40–46), EMDR group therapy significantly enhanced participants’ emotional regulation, stress tolerance, and overall psychological adjustment. These results further support the therapeutic potential of EMDR in promoting collective healing and emotional stabilization among disaster-affected adolescents (46, 47).

In the qualitative analysis, the most striking finding was the recurrent emphasis on the “reconstruction of a sense of safety and trust” in participants’ narratives. Elements such as the *“safe place exercise”* and *“family support”* played a particularly powerful role in restoring emotional stability and fostering a sense of inner security (48). This finding suggests that, in the aftermath of trauma, individuals seek not only psychological recovery but also the reestablishment of relational trust, a sense of belonging, and emotional safety. Therefore, the restoration of trust can be understood not merely as an outcome of trauma recovery but as one of its most essential and transformative components. The theme of rebuilding trust after trauma is consistent with findings from previous studies that highlight similar processes of emotional and relational restoration (40, 41, 49). Because adolescents’ coping styles are not well-developed, they can be more affected by the traumatic events they experience (50). In this case, they can often be affected by a number of psychological conditions, such as symptoms of post-traumatic stress disorder, depression, and anxiety (51). Furthermore, the meaning children attach to traumatic events plays a crucial role in the psychological effects they experience (52). In this study, the participants’ initially high levels of PTSD, depression, and anxiety were significantly reduced after the intervention, consistent with previous studies (22).

The results of this study support previous literature by demonstrating that EMDR group therapy reduces PTSD symptoms, depression, and anxiety, and positively enhances psychological resilience. These results are consistent with previous EMDR group therapies conducted with a mixed design (53–61). and also the studies conducted only with adolescents after natural disasters (40, 42, 44, 53, 57, 61) have yielded results that support this.

Adolescents exposed to natural disasters often experience high levels of psychological distress, yet access to appropriate mental health support remains limited. Filazoglu Cokluk et al. (9) emphasizes that emotional responses to trauma are often heightened during adolescence, a period characterized by vulnerability and developmental changes. (12). At the same time, these same adolescents are at an increased risk of developing comorbidities such as depression and anxiety, which can inhibit their overall development and hinder their survival mechanisms after trauma (62). Institutions providing mental health services after disasters can leverage group dynamics to instill a sense of solidarity and shared resilience, improving the overall therapeutic experience (63). Importantly, group therapy can also improve access to treatment by allowing clinicians to reach more individuals simultaneously, which is crucial in resource-limited settings that often follow catastrophic events.

Nevertheless, the effects of EMDR on certain anxiety subtypes, such as social anxiety disorder and panic disorder, were modest, with no statistically significant group differences detected in these areas. This limited impact may be linked to the brief duration of the group sessions and the more individualized nature of these specific anxiety patterns, which often require longer-term or more tailored interventions. Despite this, participants who underwent EMDR demonstrated significantly higher resilience scores, indicating enhanced psychological adaptability and improved coping abilities following treatment. There are several studies in the literature suggesting that different treatment protocols are more effective in this regard (64–68). Results from Aydın’s (69) systematic review support this finding. Longer-term methods and being directly targeted are among the criteria for achieving effective results. Aydın (69) particularly emphasized cognitive behavioral treatments. Applying EMDR protocols appropriate to the symptoms may be necessary for effective outcomes. Specifically, when the goal in this study was to reduce symptoms of post-traumatic stress disorder, depression, and anxiety, different protocols for other sub-symptoms would increase effectiveness. This situation is also supported by studies in the literature (70–72).

Overall, the findings highlight EMDR group therapy as an effective approach for reducing trauma-related stress, depression, and generalized anxiety while fostering resilience among adolescents exposed to the earthquakes. EMDR has attracted attention for its relatively quick effects and its effectiveness in diverse populations, including adolescents (62). The potential for group EMDR therapy to be implemented specifically for adolescents affected by disasters is particularly intriguing; Collective experiences of trauma can promote shared understanding and resilience among participants, reducing feelings of isolation and offering emotional and social support. Although its effects on social and panic anxiety were less pronounced, the intervention contributed meaningfully to emotional recovery and overall mental well-being in the study group.

However, implementing group EMDR therapy raises several challenges that deserve careful consideration. Potential barriers include variability in therapeutic efficacy based on individual differences, as not all adolescents may respond similarly in a group context. Furthermore, cultural factors can influence the acceptance and effectiveness of EMDR, necessitating cultural competence among therapists (62). Additionally, logistical challenges, such as finding trained clinicians, coordinating group sessions, and providing follow-up support, may further complicate the successful adoption of this intervention in post-disaster settings. Thus, although the group EMDR approach presents a valuable opportunity to improve mental health outcomes among adolescents affected by disasters, it must be implemented with an awareness of these significant barriers and a commitment to addressing them effectively., The effectiveness of EMDR therapy in alleviating symptoms of PTSD, anxiety, and depression among disaster-affected adolescents is gaining substantial empirical support. The research by Xie et al. (13) and Galvan et al. (2021) (73) demonstrate that group EMDR therapy significantly reduces the severity of PTSD, along with associated symptoms of anxiety and depression in adolescents who have experienced traumatic disasters. In controlled settings, adolescents who participated in group EMDR showed marked decreases in trauma-related distress, with follow-up assessments illustrating lasting effects that contribute to improved mental health outcomes. This is particularly noteworthy given the vulnerability of adolescents in disaster settings, where traditional treatment modalities may fail to address the specific needs of this population.

Additionally, the methodological complexities inherent in mixed methods research pose additional challenges when evaluating the impact of group EMDR therapy. The convergence of qualitative and quantitative data allows for a more nuanced understanding of participants’ experiences; however, this also complicates the analysis process. Entrepreneur et al. (33) emphasize that integrating diverse types of data requires rigorous methodological frameworks to ensure validity and reliability. The challenges of triangulating data, particularly in distinguishing subjective experiences from quantitative findings, require careful consideration of study design and analysis. This complexity can lead to potential misinterpretations of results, which can obscure the true effectiveness of therapy and hinder the advancement of evidence-based practices in disaster-affected settings. This is especially important for planning real-world implementation. Although many adolescents were open to therapy, those with the greatest trauma may need extra support, such as emotional preparation, safe and private spaces, and step-by-step guidance, to feel ready for group-based treatment.

Kaptan et al. (2021)’s (74) systematic review warns that group EMDR’s evidence of effectiveness is still limited by methodological heterogeneity, the perspective from which the current study’s naturalistic design (e.g., non-random sampling) needs to be considered (25, 75). Compared to other therapeutic approaches, group EMDR therapy has multiple advantages, particularly regarding cost-effectiveness and accessibility. Lucas and Brown highlight that group interventions leverage peer support structures, thereby reducing the financial burden on individual patients and healthcare systems. Furthermore, İkican and Mor (76) argue that group EMDR facilitates a community atmosphere where shared experiences can foster connection between participants, mitigating feelings of isolation often reported by adolescents coping with trauma. The inclusive nature of group therapy not only encourages engagement, but also allows clinicians to efficiently address the needs of multiple individuals at the same time, which is especially beneficial in low-resource settings (77)

The successful implementation of group EMDR therapy has been documented in several clinical and community settings, underscoring its versatility. Gibbs et al. illustrate how group EMDR has been effectively used in post-disaster recovery contexts, achieving positive therapeutic outcomes. Participants reported reductions in symptoms of anxiety, depression, and PTSD, which were statistically significant compared to pre-treatment assessments. Furthermore, Laksmita et al. (78) expand on this topic by documenting a multi-site initiative in which group EMDR was integrated into school-based mental health programs, demonstrating that adolescents who experienced this intervention not only showed relief from symptoms, but also showed greater coping skills and improved resilience. These studies confirm that group EMDR therapy is not simply a temporary palliative solution; is a powerful intervention that can promote long-term mental health and emotional well-being among adolescents in crisis. However, Xie et al.’s (13) meta-analysis places EMDR as the most effective disaster intervention for youth PTSD, consistent with the current study’s participants’ receptiveness to “creative approaches” (e.g., 42% preferred art-based EMDR) (13).

Whereas previous trials validate EMDR-IGTP’s effectiveness in symptom reduction following implementation (20, 25), the current study’s experiment lays bare pre-intervention obstacles: severity of PTSD suppresses and disaster settings might weaken gender/awareness impacts seen in clinical or refugee contexts. This underscores Heinz et al.’s priority of “meeting youth where they are,” either through hybrid (digital + group) formats or resilience-enhancing preludes to group treatment (79). Grolnick et al. recommend adapting therapy models to better fit the unique needs of adolescents following a disaster. This may include flexible schedules and incorporating community resources, such as schools and local organizations, for easier access. Additionally, establishing support groups within the community can help destigmatize mental health treatment and normalize the conversation about emotional difficulties. Yıldız et al. (80) suggest peer support initiatives that empower adolescents to share their experiences, thereby fostering a supportive environment that encourages participation in therapeutic modalities such as EMDR. these findings highlight that while EMDR-IGTP is generally acceptable to adolescents, careful attention must be given to the emotional and practical needs of those with higher trauma levels. By combining flexible delivery, creative tools, and early psychoeducation, group therapy can become more accessible and effective for disaster-affected youth.

While this study highlights the effectiveness of EMDR group therapy in reducing trauma symptoms and enhancing resilience, it is important to compare it with other psychosocial interventions. Cognitive Behavioral Therapy (CBT) has been shown to effectively decrease PTSD and depression symptoms among adolescents after disasters (64, 65, 81, 82). However, unlike EMDR—which directly targets traumatic memories—CBT usually requires longer treatment and greater cognitive effort, which may challenge highly traumatized adolescents (67, 68). Mindfulness and supportive counseling also improve emotional regulation (62, 66), though their effects are often slower and less focused on trauma processing. EMDR’s rapid and embodied techniques appear to provide faster stabilization, but integrating it with CBT or psychoeducation could yield more comprehensive and lasting recovery outcomes (69, 79).

Furthermore, the need for consistent follow-up and longitudinal studies is highlighted as essential to capture the lasting effects of group EMDR therapy. Figley (34) argues that immediate post-intervention assessments may not fully reflect the long-term benefits or harms of therapy, particularly in populations with complex trauma histories. By establishing follow-up protocols, researchers can monitor the durability of therapeutic effects over time and identify emerging barriers that may hinder continued access to mental health support. Longitudinal studies can provide invaluable information about participants mental health trajectories post-intervention, thereby informing future therapeutic strategies and policies.

## Strengths and limitations

### Strengths

This study uses a convergent mixed methods design to assess PTSD severity, resilience, and anxiety in adolescents. It combines quantitative assessment with qualitative understanding of facilitators and barriers to EMDR IGTP. The study also explores pre-treatment acceptability, a crucial component for effective post-disaster application.

### Limitations

The study has several methodological concerns, including potential response biases due to self-reported questionnaires, a cross-sectional nature that only captures pre-intervention perceptions without observing their development post-intervention, and the potential for results to be generalizable to other adolescents in different settings due to natural disasters. Thematic analysis, while providing contextual information, could benefit from triangulation of observational or facilitator-reported data to enhance credibility. Coding qualitative data carries a risk of subjective interpretation. Using more coders or software-assisted analysis (e.g., NVivo) in future studies may yield more effective results. Coding qualitative data carries a risk of subjective interpretation. Using more coders or software-assisted analysis (e.g., NVivo) in future studies is recommended.

While this study provides important insights into adolescents’ pre-intervention perceptions, it lacks post-intervention outcome data. The absence of follow-up measures on PTSD symptoms and resilience limits our ability to assess the therapeutic impact of EMDR-IGTP. Future studies should adopt a pre–post design using the same instruments—specifically, the CPSS-5-R and CD-RISC-10—to evaluate symptom changes following EMDR-IGTP participation. Including longitudinal outcome data would strengthen conclusions regarding the intervention’s effectiveness and sustainability. Another critical direction for future research is the inclusion of longitudinal follow-up assessments at 3 to 6 months post-intervention. Tracking long-term changes in PTSD symptoms and resilience would help establish the sustained efficacy of EMDR-IGTP and identify whether initial feasibility perceptions translate into lasting therapeutic engagement and symptom reduction.

### Future recommendations

The extension of data collection to representative cultural and socioeconomic environments and the incorporation of objective indicators (e.g., attendance at sessions, behavioral engagement measures) will further maximize generalizability and guide scalable, trauma-informed implementation approaches for adolescents in post-disaster environments. Future implementations of EMDR-IGTP should incorporate structured psychoeducational modules to prepare adolescents for group therapy. These may include brief animated videos explaining the phases of EMDR, illustrated trauma-recovery booklets tailored to adolescents, and pre-session orientation workshops. Participants in the current study emphasized the need for clear explanations and emotional readiness, suggesting that such resources could reduce anxiety, demystify the process, and increase participation among adolescents unfamiliar with therapy.

## Data Availability

The original contributions presented in the study are included in the article/supplementary material. Further inquiries can be directed to the corresponding author.
